# Liver Oxidative Status, Serum Lipids Levels after Bariatric Surgery and High-Fat, High-Sugar Diet in Animal Model of Induced Obesity

**DOI:** 10.3390/ijms242216535

**Published:** 2023-11-20

**Authors:** Wojciech Kazura, Katarzyna Michalczyk, Bronisława Skrzep-Poloczek, Elżbieta Chełmecka, Jolanta Zalejska-Fiolka, Marek Michalski, Michał Kukla, Jerzy Jochem, Jakub Rutkowski, Dominika Stygar

**Affiliations:** 1 Department of Physiology, Faculty of Medical Sciences in Zabrze, Medical University of Silesia, 19 Jordana Street, 41-808 Zabrze, Poland; 2 Department of Medical Statistics, Faculty of Pharmaceutical Sciences in Sosnowiec, Medical University of Silesia, 31 Ostrogórska Street, 41-200 Sosnowiec, Poland; 3 Department of Biochemistry, Faculty of Medical Sciences in Zabrze, Medical University of Silesia, 19 Jordana Street, 41-808 Zabrze, Poland; 4 Department of Histology, Faculty of Medical Sciences in Zabrze, Medical University of Silesia, 19 Jordana Street, 41-808 Zabrze, Poland; 5 Department of Internal Medicine and Geriatrics, Faculty of Medicine, Jagellonian University Medical College, 31-688 Kraków, Poland; 6 Department of Endoscopy, University Hospital, 30-688 Kraków, Poland; 7 SLU University Animal Hospital, Swedish University of Agricultural Sciences, SE-750 07 Uppsala, Sweden

**Keywords:** bariatric surgery, duodenojejunal omega switch, high-fat, high-sugar diet, lipids, liver, oxidative stress

## Abstract

Nutritional status is a major determinant of hepatocyte injuries associated with changed metabolism and oxidative stress. This study aimed to determine the relations between oxidative stress, bariatric surgery, and a high-fat/high-sugar (HFS) diet in a diet-induced obesity rat model. Male rats were maintained on a control diet (CD) or high-fat/high-sugar diet (HFS) inducing obesity. After 8 weeks, the animals underwent SHAM (*n* = 14) or DJOS (*n* = 14) surgery and the diet was either changed or unchanged. Eight weeks after the surgeries, the activity of superoxide dismutase isoforms (total SOD, MnSOD, and CuZnSOD), catalase (CAT), glutathione peroxidase (GPx), glutathione reductase (GR), and lutathione S-transferase, as well as the thiol groups (-SH) concentration, total antioxidant capacity (TAC), total oxidative stress (TOS) levels, and malondialdehyde (MDA) concentration liver tissue were assessed. The total cholesterol, triglycerides (TG), and high-density lipoprotein (HDL) concentrations were measured in the serum. The total SOD and GPX activities were higher in the SHAM-operated rats than in the DJOS-operated rats. The MnSOD activity was higher in the HFS/HFS than the CD/CD groups. Higher CuZnSOD, GST, GR activities, -SH, and MDA concentrations in the liver, and the triglyceride and cholesterol concentrations in the serum were observed in the SHAM-operated rats than in the DJOS-operated rats. The CAT activity was significantly higher in the HFS-fed rats. Lower TAC and higher TOS values were observed in the SHAM-operated rats. Unhealthy habits after bariatric surgery may be responsible for treatment failure and establishing an obesity condition with increased oxidative stress.

## 1. Introduction

Oxidative stress is a significant element in pathological obesity regarded as a severe health burden worldwide due to its related comorbidities, such as diabetes, cardiovascular disease, hepatic steatosis, and non-alcoholic fatty liver disease (NAFLD) [1]. Oxidative stress generates reactive oxygen species (ROS), which activate enzymatic and non-enzymatic antioxidant systems, neutralizing them. According to clinical and experimental investigations, these antioxidant systems are altered during NAFLD development [2].

Aside from long-term weight loss, bariatric surgery helps to manage metabolic processes, resulting in improved glucose tolerance and insulin sensitivity and increased energy expenditure [3]. As described previously [4,5,6], a duodenal–jejunal omega switch (DJOS) is a bariatric procedure using a proximal duodeno-enterostomy loop to bypass the foregut and allow for direct hindgut stimulation. The animal model used allows for addressing questions regarding the effects of DJOS that could not be pursued experimentally in humans otherwise [4,5,6]. Dietary patterns greatly contribute to the genesis and risk factors of such diseases as metabolic syndrome, NAFLD, and obesity. A high-fat/high-sugar (HFS) diet disrupts the expression of inflammatory markers and can impair adaptive immunity in obese people. The HFS diet is known to cause hypertension, reduced glucose tolerance, increased abdominal fat deposition and abdominal circumference, and an altered lipid profile [7]. The digestion of different types of dietary products has different effects on the oxidative status of the organism. Nutritional status is a major determinant of hepatocyte injuries associated with changed metabolism and oxidative stress [8].

The present study aimed to determine the effects and relations between oxidative stress, bariatric surgery, and a high-fat/high-sugar (HFS) diet in a diet-induced obesity (DIO) rat model.

## 2. Results

### 2.1. Antioxidant Status Markers

**Figure 1:**
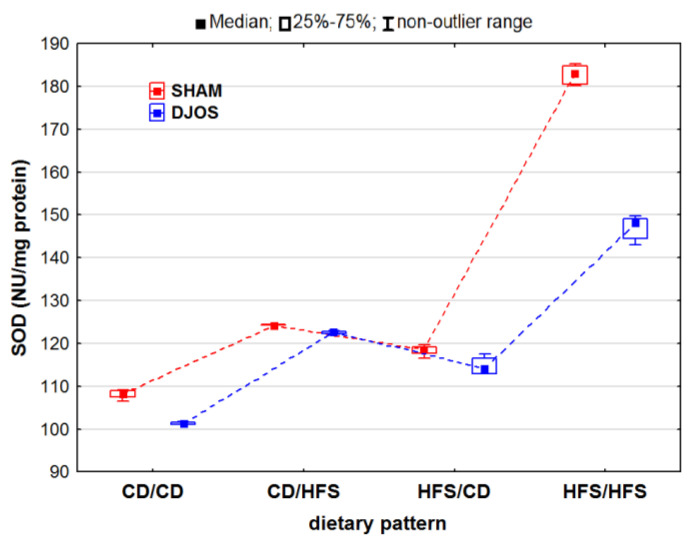
Total superoxide dismutase (SOD) activity (NU/mg protein) in the livers of Sprague-Dawley rats (*n* = 56) after duodenojejunal omega switch (DJOS, *n* = 28,) or control (SHAM, *n* = 28) surgery; rats were fed according to different dietary patterns (CD/CD, *n* = 7; CD/HFS, *n* = 7; HFS/CD, *n* = 7; HFS/HFS, *n* = 7) for 8 weeks after the surgery. Legend: CD—control diet, HFS—high-fat, high-sugar diet. For reader’s convenience, the dashed lines connect the groups subjected to the same type of surgery.

We noted statistically significant differences in the total SOD activity in both the SHAM- and DJOS-operated animals (p_SHAM_ < 0.001, p_DJOS_ < 0.001). The total SOD activity in the livers of the SHAM-operated rats was higher, especially in the rats fed with the same diet (CD/CD and HFS/HFS) during the whole experiment. In the case of these rats, we found statistically significant differences in the total SOD activity with higher values noted for the rats fed with the HFS diet only compared to the rats fed with the CD diet only (p_SHAM_ < 0.05, p_DJOS_ < 0.001). No differences in the total SOD activity were noted in the livers of the rats fed according to the changing dietary pattern (Figure 1).

**Figure 2:**
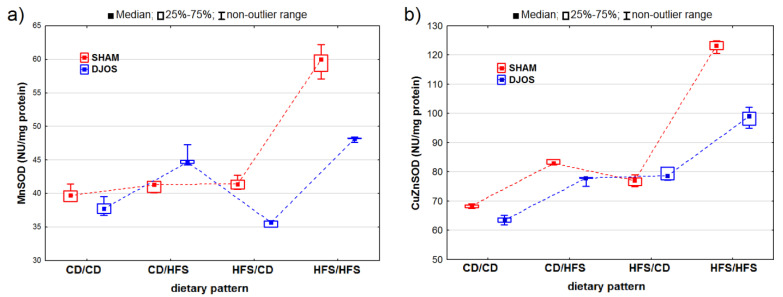
Mn–superoxide dismutase (MNSOD) activity (NU/mg protein) (**a**) and Cu–Zn–superoxide dismutase (CuZnSOD) activity (NU/mg protein) (**b**) in the livers of Sprague-Dawley rats (*n* = 56) after duodenojejunal omega switch (DJOS, *n* = 28,) or control (SHAM, *n* = 28) surgery; rats were fed according to different dietary patterns (CD/CD, *n* = 7; CD/HFS, *n* = 7; HFS/CD, *n* = 7; HFS/HFS, *n* = 7) for 8 weeks after the surgery. Legend: CD—control diet, HFS—high-fat, high-sugar diet. For reader’s convenience, the dashed lines connect the groups subjected to the same type of surgery.

We identified statistically significant changes in the MnSOD activity of the liver tissue of rats from the DJOS- and SHAM-operated groups (p_DJOS_ < 0.001, p_SHAM_ < 0.001). Apart from the rats fed with the CD/HFS dietary pattern, we identified higher MnSOD activity in the SHAM-operated groups (Figure 2A). We observed statistically significant changes in the MnSOD activity in the groups fed with the same diet before and after the surgery, with higher MnSOD activity in the HFS/HFS groups compared to the CD/CD groups (p_DJOS_ < 0.05, p_SHAM_ < 0.001). The MnSOD activity in the livers of the SHAM-operated rats fed with the CD/CD, CD/HFS, and HFS/CD dietary pattern was the same.

We discovered statistically significant differences in the CuZnSOD activity between the DJOS- and SHAM-operated rats (p_DJOS_ < 0.001, p_SHAM_ < 0.001). We found higher CuZnSOD activity in the SHAM-operated rats fed according to all the dietary patterns, except for the HFS/CD pattern (Figure 2B). We detected statistically significant changes in the CuZnSOD activity in the groups of rats fed with the same diet during the experiment, with higher CuZnSOD activity in the livers of rats from the HFS/HFS group than in the CD/CD group (p_DJOS_ < 0.001, p_SHAM_ < 0.001). We observed no statistically significant changes in the SHAM- (*p* = 0.849) and DJOS-operated animals (*p* = 1) from the groups that had the diet changed after the surgery.

**Figure 3:**
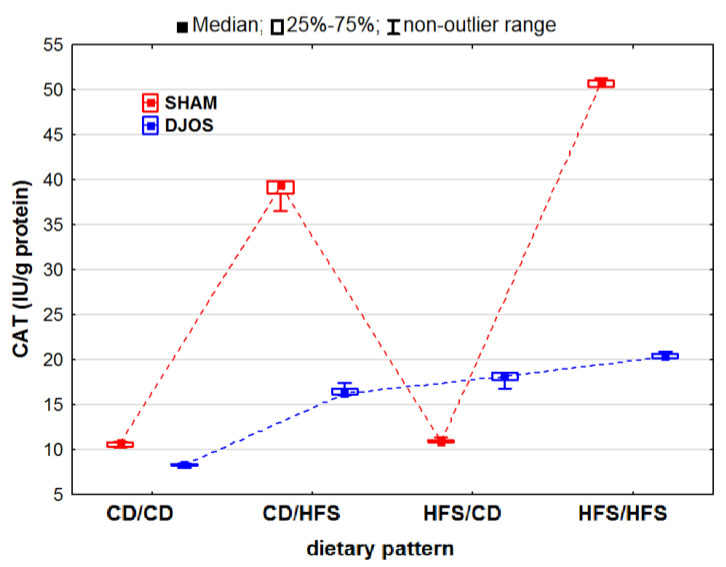
Catalase (CAT) activity (IU/g protein) in the livers of Sprague-Dawley rats (*n* = 56) after duodenojejunal omega switch (DJOS, *n* = 28,) or control (SHAM, *n* = 28) surgery; rats were fed according to different dietary patterns (CD/CD, *n* = 7; CD/HFS, *n* = 7; HFS/CD, *n* = 7; HFS/HFS, *n* = 7) for 8 weeks after the surgery. Legend: CD—control diet, HFS—high-fat, high-sugar diet. For reader’s convenience, the dashed lines connect the groups subjected to the same type of surgery.

We discovered statistically significant differences in the CAT activity in the DJOS- and SHAM-operated rats (p_DJOS_ < 0.001, p_SHAM_ < 0.001). However, we observed decreased CAT activity in the SHAM-operated rats compared to the DJOS-operated rats, but only in the rats fed according to the HFS/CD dietary pattern (Figure 3). For the DJOS-operated animals, the lowest CAT activity was noted for the animals fed according to the CD/CD dietary pattern, but it did not differ from the CAT activity noted for the animals fed according to the CD/HFS (*p* = 0.053) or HFS/CD (*p* = 1) dietary pattern. When comparing the CAT activity in the rats fed according to the mixed dietary pattern (CD/HFS vs. HFS/CD), we observed no statistically significant differences in either the DJOS- (p_DJOS_ = 0.990) or SHAM-operated animals (p_SHAM_ = 0.434). In the rats fed with the same diet before and after the surgery (CD/CD and HFS/HFS), the CAT activity was significantly higher in the animals fed with the HFS diet during the whole experiment (p_DJOS_ < 0.001, p_SHAM_ < 0.001) (Figure 3).

**Figure 4:**
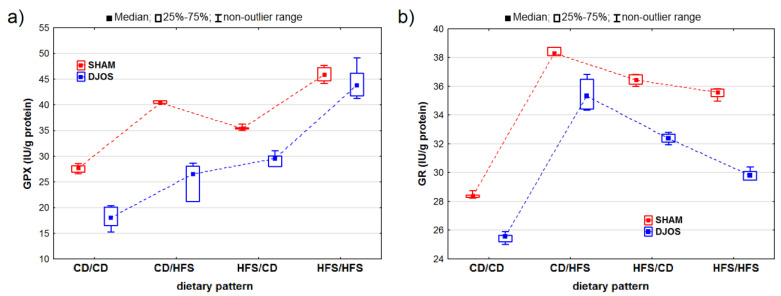
Glutathione peroxidase (GPx) activity (IU/g protein) (**a**) and glutathione reductase (GR) activity (IU/g protein) (**b**) in the livers of Sprague-Dawley rats (*n* = 56) after duodenojejunal omega switch (DJOS, *n* = 28,) or control (SHAM, *n* = 28) surgery; rats were fed according to different dietary patterns (CD/CD, *n* = 7; CD/HFS, *n* = 7; HFS/CD, *n* = 7; HFS/HFS, *n* = 7) for 8 weeks after the surgery. Legend: CD—control diet, HFS—high-fat, high-sugar diet. For reader’s convenience, the dashed lines connect the groups subjected to the same type of surgery.

The DJOS and SHAM surgeries had statistically significant effects on changes in the GPX activity (p_DJOS_ < 0.001, p_SHAM_ < 0.001). We observed higher GPX activity in the SHAM-operated animals, irrespective of the used dietary pattern (Figure 4A). We noted statistically significant changes in the GPX activity of the rats fed with the same diet before and after the surgery (HFS/HFS and CD/CD), with higher GPX activity in the rats fed only with the HFS diet than in the rats fed with the control diet (pDJOS < 0.001, p_SHAM_ < 0.001). No statistically significant differences in the GPx activity were observed in the rats with a change in diet after the surgery (p_SHAM_ = 0.849, p_DJOS_ = 1) (Figure 3).

Both the DJOS and SHAM surgeries had statistically significant effects on the GR activity (p_DJOS_ < 0.001, p_SHAM_ < 0.001). In the DJOS-operated rats, the ones fed according to the CD/HFS and HFS/CD dietary patterns showed the highest levels of GR activity. The GR activity was reduced in all the groups of the DJOS-operated rats, irrespective of the dietary pattern followed during the experiment compared to the SHAM-operated rats. The rats fed with the same diet before and after surgery (CD/CD and HFS/HFS) showed no statistically significant difference in GR activity (p_DJOS_ = 0.849, p_SHAM_ = 0.849). However, the rats fed only with the control diet (CD/CD) presented the lowest GR activity in both the DJOS- and SHAM-operated groups. The rats that had their diet changed after the surgery showed higher GR activity, but no statistically significant differences were observed between the rats fed according to the CD/HFS and HFS/CD dietary patterns (Figure 4B).

**Figure 5:**
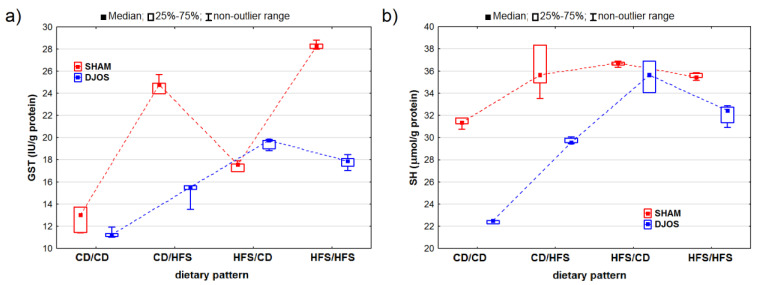
Glutathione S-transferase (GST) activity (IU/g protein) (**a**) and sulfhydryl group (SH) concentration (μmol/g protein) (**b**) in the livers of Sprague-Dawley rats (*n* = 56) after duodenojejunal omega switch (DJOS, *n* = 28,) or control (SHAM, *n* = 28) surgery; rats were fed according to different dietary patterns (CD/CD, *n* = 7; CD/HFS, *n* = 7; HFS/CD, *n* = 7; HFS/HFS, *n* = 7) for 8 weeks after the surgery. Legend: CD—control diet, HFS—high-fat, high-sugar diet. For reader’s convenience, the dashed lines connect the groups subjected to the same type of surgery.

Both the DJOS and SHAM-operated rats showed statistically significant changes in GST activity (p_DJOS_ < 0.001, p_SHAM_ < 0.001). The SHAM-operated rats presented higher GST activity, except for the rats fed according to the HFS/CD dietary pattern (Figure 5A). We observed statistically significant differences in the GST activity between the rats fed according to different dietary patterns, with higher GST activity in the rats fed with the HFS diet than in the rats fed with the control diet during the whole experiment (HFS/HFS vs. CD/CD; p_DJOS_ < 0.05, p_SHAM_ < 0.001). No statistically significant differences (*p* = 0.849) were noted in the SHAM-operated rats that had the diet changed after the surgery. However, such differences were observed in the DJOS-operated rats only (*p* < 0.05) (Figure 5A).

We noted statistically significant differences in the SH group concentrations in both the DJOS- and SHAM-operated rats (p_DJOS_ < 0.001, p_SHAM_ < 0.01). The SH group concentration was lower in the DJOS-operated rats (Figure 5B). The lowest SH group concentrations were measured in the rats fed with the control diet only (CD/CD) in both the DJOS- and SHAM-operated rats. Furthermore, the concentration of the SH groups did not differ statistically between the rats fed the same diet (CD/CD and HFS/HFS) before and after the surgery in both study groups (p_DJOS_ = 0.849, p_SHAM_ = 0.849). The concentration of the SH groups was higher in the rats with the changed dietary pattern (CD/HFS and HFS/CD), but no statistically significant differences were noted between them. The lowest concentrations of the SH groups were noted for the rats fed with the control diet only (CD/CD). In the DJOS-operated rats, they were statistically lower than in the rats fed with HFS diet only (*p* < 0.05), while in the SHAM-operated rats, the concentrations were higher (*p* = 0.164).

**Figure 6:**
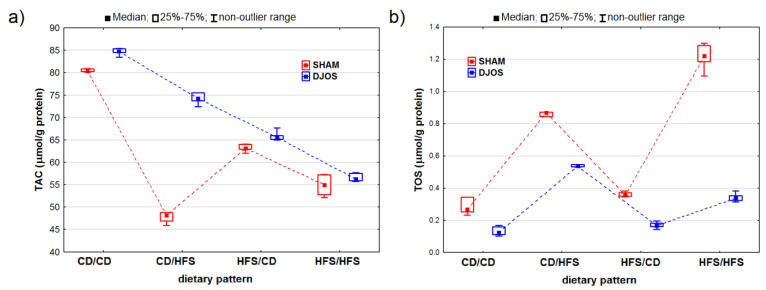
Total antioxidant concentration (TAC) (μmol/g protein) (**a**) and total oxidative status (TOS) (μmol/g protein) (**b**) in the livers of Sprague-Dawley rats (*n* = 56) after duodenojejunal omega switch (DJOS, *n* = 28,) or control (SHAM, *n* = 28) surgery; rats were fed according to different dietary patterns (CD/CD, *n* = 7; CD/HFS, *n* = 7; HFS/CD, *n* = 7; HFS/HFS, *n* = 7) for 8 weeks after the surgery. Legend: CD—control diet, HFS—high-fat, high-sugar diet. For reader’s convenience, the dashed lines connect the groups subjected to the same type of surgery.

We detected statistically significant variations in the TAC levels among the rats assigned to different dietary groups in both the DJOS- and SHAM-operated rats (pDJOS < 0.001, pSHAM < 0.001). We observed lower TAC values in the livers of the SHAM-operated rats from all the dietary pattern groups (Figure 6A). We noted statistically significant differences in the TAC levels between the rats fed with the control (CD/CD) and HFS (HFS/HFS) diet only, with lower values noted for the rats fed with the HFS diet (pDJOS < 0.001, pSHAM < 0.05). We found statistically significant differences in the TAC levels in the SHAM-operated rats that had the diet changed after the surgery (*p* < 0.05) and no such differences between the respective dietary pattern groups for the DJOS-operated rats.

We observed statistically significant differences in the TOS levels in both the DJOS- and SHAM-operated rats (p_DJOS_ < 0.001, p_SHAM_ < 0.001). The SHAM-operated rats from all the dietary groups presented higher TOS levels (Figure 6B). We discovered statistically significant higher TOS levels in the rats fed with the HFS diet only compared to the rats fed with the control diet only (p_DJOS_ < 0.05, p_SHAM_ < 0.001). The DJOS-operated rats showed changes in the TOS levels, but only the groups that had the diet changed after the surgery (*p* < 0.05). No such changes were observed in the SHAM-operated rats.

**Figure 7:**
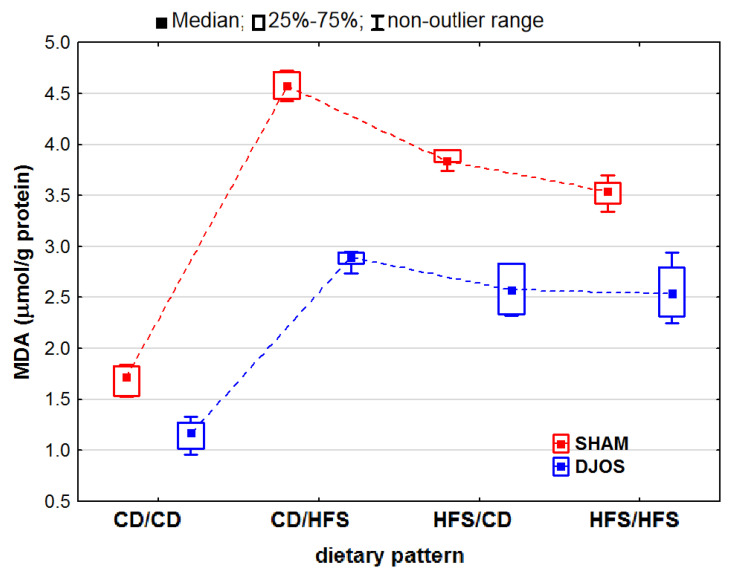
Malondialdehyde (MDA) concentration (μmol/g protein) in the livers of Sprague-Dawley rats (*n* = 56) after duodenojejunal omega switch (DJOS, *n* = 28,) or control (SHAM, *n* = 28) surgery; rats were fed according to different dietary patterns (CD/CD, *n* = 7; CD/HFS, *n* = 7; HFS/CD, *n* = 7; HFS/HFS, *n* = 7) for 8 weeks after the surgery. Legend: CD—control diet, HFS—high-fat, high-sugar diet. For reader’s convenience, the dashed lines connect the groups subjected to the same type of surgery.

We noted statistically significantly differences in the MDA concentrations in the livers of the rats subjected to both DJOS and SHAM surgery (p_DJOS_ < 0.01, p_SHAM_ < 0.001). We found that the SHAM-operated rats from all the dietary pattern groups presented increased MDA concentrations. We detected statistically significant differences in the MDA concentrations in both the unchanged (p_DJOS_ = 0.119, p_SHAM_ = 0.849) or modified diet groups (Figure 7).

### 2.2. Cholesterol, Triglyceride (TG), and HDL Levels in the Serum

We detected that in both the DJOS- (*p* < 0.001) and SHAM-operated rats, the cholesterol levels differed significantly depending on the followed dietary patterns (Table 1). The rats fed only with the control diet (CD/CD group) had the lowest, while those fed only with the HFS diet (HFS/HFS) had the highest cholesterol levels. We discovered no statistically significant differences between the rats fed according to the CD/CD and CD/HFS or CD/HFS and HFS/HFS dietary patterns in the DJOS-operated rats (Table 2). We found that the cholesterol levels differed between the SHAM-operated rats fed according to the CD/CD and CD/HFS and between the CD/CD and HFS/HFS dietary patterns (Table 2). The DJOS-operated rats presented lower cholesterol levels than the SHAM-operated rats (Table 1).

We found that the rats subjected to DJOS surgery presented lower levels of triglycerides (TG) than the rats submitted to SHAM surgery. We discovered statistically significant changes in the TG concentrations among the groups fed according to different dietary patterns when looking at each type of surgery independently (*p* < 0.001). In the DJOS- and SHAM-operated rats, we identified differences in the TG levels between the rats fed according to the CD/CD and CD/HFS (p_DJOS_ < 0.01, p_SHAM_ < 0.01) and the CD/CD and HFS/HFS dietary patterns (both *p* < 0.001). Higher TG concentrations were observed in the rats fed with the HFS diet (CD/HFS and HFS/HFS) after each type of surgery, and the concentrations did not differ significantly among these groups (Table 2).

As for HDL, the SHAM-operated rats presented reduced HDL concentrations, except for the rats fed according to the HFS/CD dietary pattern. We identified statistically significant changes in the HDL levels based on the applied dietary pattern in the rats subjected to SHAM or DJOS surgery (Table 1). The HDL concentrations differed between the rats fed only with the control diet (CD/CD) and those fed according to the HFS/CD dietary pattern (Table 2).

## 3. Discussion

Despite the fact that oxidative stress is an imminent characteristic of diet-induced obesity (DIO), no single biomarker has been identified as a gold standard reflecting the redox state of this metabolic illness so far [9]. Since different studies report different antioxidant levels/activities in the DIO context, the present study aimed to assess the enzymatic and non-enzymatic oxidative stress markers in the liver tissue, as oxidative stress is thought to be the primary cause of liver damage and liver-related disease development, such as NAFLD [10,11]. Non-alcoholic fatty liver disease is the most prevalent chronic hepatic pathology, and the clinical spectrum includes non-alcoholic simple steatohepatitis (NASH) or cirrhosis with all the characteristics of portal hypertension, simple hepatic steatosis, and, subsequently, hepatic cell carcinoma (HCC) [12]. According to some research, oxidative stress and the renin–angiotensin system (RAS) may mediate liver injury during NAFLD [13]. In alcoholic liver disease (ALD), pathogenesis appears to begin with elevated angiotensin II levels. A study on Ren2 transgenic rats shows that elevated levels of endogenous angiotensin II had pro-oxidant, pro-inflammatory, and pro-fibrotic effects in the liver. In addition, the elevated levels of angiotensin II induced ROS production in the liver [14,15].

Oxidative stress is particularly damaging to mitochondria, as it lowers the number of mitochondria, impairs mitochondrial gene expression and proteins synthesis, and affects beta-oxidation [12]. Elevated NADPH oxidase activity and reduced CuZnSOD activity are signs of increased ROS production during ALD. Excessive oxidative agent production causes lipid peroxidation, which additionally enhances mitochondrial permeability and modifies their function [16,17].

Exogenous factors that promote oxidative stress in liver physiopathology include diet, viruses, alcohol, and medications. Endogenous factors include insulin resistance, obesity, and diabetes [18]. The present study demonstrates that adherence to the HFS diet combined with bariatric surgery affected the liver oxidative status and blood lipid profiles in rats with DIO. This study reveals that DJOS surgery combined with HFS consumption for eight weeks before and after the surgery led to significant reductions in the liver CAT, SOD, MnSOD, CuZnSOD, GPX, GR, and GST activities, and SH groups, TOS, TAC, and MDA levels in all the DJOS-operated rats compared to the SHAM-operated rats. Increased oxidative stress undoubtedly plays a crucial role in the development of numerous chronic liver diseases and stimulates their progression. Oxidative stress underlies viral and alcoholic liver diseases, participates in the liver fibrogenic response, and results in altered gene expression and progressive liver damage [19,20,21]. A similar result was observed in the presented study. The serum lipid profiles, such as the total cholesterol and TG, were lower in the DJOS-operated groups regardless of the type of diet consumed before and after the surgery.

The primary cause of oxidative stress occurring in the liver is the detoxifying process itself, as one of the most essential functions of the liver is filtering the blood and neutralizing potentially harmful exo- or endogenous substances [22]. Oxidation and detoxification processes of metabolites, secondary compounds, and toxins generate reactive oxygen species (ROS) as intermediate compounds of biotransformation processes [12]. An excess of metabolites or lack of natural antioxidants promotes oxidative stress, causing tissue damage and inflammation, which seems to be one of the most critical pathogenic processes during DIO development and a link between simple steatosis and non-alcoholic simple steatohepatitis (NASH) expression [18,23]. Many chronic illnesses, particularly those characterized by low-grade inflammation, such as diabetes, metabolic syndrome, and obesity, relate to oxidative stress [23].

The first line of defense against ROS comprises the inactivating or scavenging antioxidant enzymes, such as SOD and CAT. Superoxide dismutase is the only enzyme that breaks down superoxide radicals. This process generates H_2_O_2_, which is eliminated by catalase, whose role is to inhibit the creation of hydroxyl radicals. Glutathione peroxidase also catalyzes the reduction of H_2_O_2_ and other hydroperoxides to H_2_O by oxidizing the reduced glutathione (GSH) into glutathione disulfide (GSSH) [24]. Glutathione reductase is an NADPH-dependent oxidoreductase catalyzing the conversion of the oxidized glutathione (GSSG) back to is to reduced form (GSH). Some glutathione transferase (GST) enzymes present GSH-dependent thiol transferase activity, enabling antioxidant (i.e., ascorbate, flavonoids, quinones) recycling, while other isoenzymes use GSH as co-substrate. A significant portion of GST isoenzymes also demonstrates glutathione peroxidase activity and can convert lipid peroxides and other peroxides to less harmful compounds [25]. In our study, the activities of all the enzymes analyzed in the livers of the DJOS-operated rats fed only with the HFS diet or according to mixed dietary patterns were significantly reduced compared to the SHAM-operated rats. This suggests that bariatric surgery increases the non-enzymatic antioxidant response in the liver, lowering the endogenous enzymatic defense even in the presence of the HFS diet. This study shows that the high-fat, high-sugar dietary pattern applied after DJOS surgery leads to increased MDA and reduced levels of antioxidant factors. Other studies show that patients with cirrhosis present increased levels of pro-oxidant markers, e.g., serum MDA and reduced levels of antioxidant factors, e.g., catalase, SOD, and reduced blood GSH, indicating that oxidative stress is known to factor into cirrhosis development [26].

TAC tests estimate the antioxidant components globally, whereas using individually assessed antioxidant markers is time-consuming and labor-intensive and requires complicated and expensive procedures [27,28,29]. Other benefits of employing TAC assays include the ease of use of the procedures, the cheap cost per sample, the speed of the reactions, and the ability to run them using automated, semi-automated, or manual methods [29]. In this study, the TAC was significantly higher in the DJOS-operated animals regardless of the applied dietary pattern. Nevertheless, the rats fed with HFS only presented significantly reduced TAC levels, showing that the HFS diet has deleterious effects on beneficial aspects of bariatric therapy shortly after the surgery. In addition, the oxidative damage to lipids and proteins in the rats’ livers measured with MDA was lower in the DJOS-operated rats. However, the highest levels of MDA were observed in the rats that had the diet changed to an HFS diet after the surgery (CD/HFS), suggesting that conversion form control to an HFS diet has a more deleterious impact on lipid peroxidation than consuming an HFS diet before and after DJOS surgery. The mechanism of this phenomena needs further investigation.

Excessive lipids deposited in visceral adipose tissue and in fatty liver negatively influence the antioxidant capacity and increase ROS generation [30,31]. In this study, we observed that the rats subjected to the DJOS surgery presented reduced serum cholesterol and TG levels. Our previous findings revealed a significant increase in the body weight of animals fed with HFS (vs. control diet) in both the DJOS- and SHAM-operated groups [7]. Hence, higher oxidation levels, measured by oxidative stress enzymes in the liver tissues of rats fed with an HFS diet, may be strongly related to the measured higher serum lipid levels and previously reported higher body weight [7].

## 4. Materials and Methods

### 4.1. Ethical Permission

The procedures within the experimental design were approved by the Ethical Committee for Animal Experimentation of the Medical University of Silesia (Katowice, Poland) (58/2014). The institutional and national guidelines for animal care and use (Directive 2010/63/EU) were followed. The number of animals used in the study was minimal, as the 3Rs guidelines for the humane treatment of animals suggest [32]. The survival rate of the animals subjected to the surgeries was 100%.

### 4.2. Study Subject

The study included 7-week-old male Sprague-Dawley rats (Charles River Breeding Laboratories, Wilmington, MA, USA) weighing 250–275 g. The rats (*n* = 56) were kept in fully controlled conditions: 12/12 h of dark/light cycle, at 23 °C, water and food ad libitum.

### 4.3. Study Design

After a 7-day acclimation, the animals were randomly divided into the experimental groups. The first part of the experiment assumed to feed the rats for 8 weeks with either a control diet (CD) (*n* = 28) or a high-fat/high-sugar diet (HFS) (*n* = 28) that induced obesity. The nutritional and energetical characteristics of the CD and HFS diets are described in detail in Stygar et al. [7].

After 8 weeks, rats from each experimental group (CD and HFS) were randomly subjected to either control (SHAM) (*n* = 14) or duodenojejunal omega switch (DJOS) (*n* = 14) surgery, creating four subgroups: CD/SHAM, CD/DJOS, HFS/SHAM, and HFS/DJOS. After the surgery, rats from each subgroup were fed either the same diet or a changed diet for an additional 8 weeks, creating eight final cohorts of 7 individuals (CD/SHAM/CD *n* = 7, CD/DJOS/CD *n* = 7, HFS/SHAM/HFS *n* = 7, HFS/DJOS/HFS *n* = 7 and CD/SHAM/HFS *n* = 7, CD/DJOS/HFS *n* = 7, HFS/SHAM/CD *n* = 7, HFS/DJOS/CD, *n* = 7, respectively). No animal deaths occurred before the study ending points.

Eight weeks after the DJOS and SHAM surgeries, the antioxidant status markers in the liver tissue and serum samples were analyzed. In the liver tissue, we analyzed the enzymatic activities of the total, Mn, and CuZn superoxide dismutase (SOD, MnSOD, and CuZnSOD), catalase (CAT), glutathione peroxidase (GPx), glutathione reductase (GR), and glutatione S-transferase. Additionally, we analyzed the non-enzymatic antioxidant levels, such as the thiol groups (-SH) concentration, total antioxidant capacity (TAC), total oxidative stress (TOS) levels, and oxidative stress index (OSI) value and the product of the lipid peroxidation–malondialdehyde (MDA) concentration. In the serum, we analyzed the total cholesterol, triglycerides (TG), and high-density lipoprotein (HDL) concentrations.

### 4.4. Experimental Procedures

#### 4.4.1. Control (SHAM) and Duodenojejunal Omega Switch (DJOS) Surgery

The animals were subjected to anesthesia with 2% isoflurane (AbbVie, Ludwigshafen, Germany) and oxygen 2 L/min under spontaneous breathing, analgesia with xylazine (5 mg/kg, ip; Xylapan, Vetoquinol Biovet, Puławy, Poland), and antibiotic prophylaxis with gentamicin (gentamycin 40 mg/mL, Krka, Warsaw, Poland). The control (SHAM) and duodenojejunal omega switch (DJOS) surgeries were performed as described by Stygar et al. [4].

The analgesia was maintained in the post-operative period for 72 h with carprofen (4 mg/kg, sc; Rimadyl, Pfizer, Zürich, Switzerland).

#### 4.4.2. Sample Collection and Preparation

Blood (700 μL) was collected via the right tail vein, which was cannulated with a 26-gauge cannula into EDTA-containing (7.2 mg) tubes (Sigma-Aldrich, Burlington, MA, USA), and then it was centrifuged (4000× *g* rpm, 10 min, 4 °C). The separated serum was collected, snap-frozen in liquid nitrogen, and stored at −80 °C until further analysis.

Liver tissues were sampled from animals that were euthanized according to the procedures approved by the Ethical Committee for Animal Experimentation of the Medical University of Silesia (Katowice, Poland). The tissues were collected in 0.9% NaCl to obtain 10% homogenates. The homogenates were centrifuged (12,000× *g*, 10 min, 4 °C), then the supernatant was collected, snap-frozen in liquid nitrogen, and stored at −80 °C until further analysis.

The protein concentration in both the liver and serum samples was determined using the Lowry method and a calibration curve prepared with bovine serum albumin as the standard [33].

#### 4.4.3. Antioxidant Status Markers Analysis

Antioxidant status marker analyses were performed using colorimetric methods. The absorbance or absorbance changes were registered using a Victor X3 reader (PerkinElmer, Inc., Waltham, MA, USA).

##### Superoxide Dismutase (SOD) (EC 1.15.1.1) Activity

The SOD activities were determined using the Oyanagui method [34]. The CuZnSOD activity was calculated from the difference between the total SOD and MnSOD activity after inhibiting the CuZnSOD activity with potassium cyanide (KCN). The SOD activities were expressed in NU/mg of protein, with 1 NU (nitrate unit) equal to 50% inhibition of nitrite ion formation.

##### Catalase (CAT) Activity (EC 1.11.1.6)

The CAT activity was determined using the Aebi method [35] and expressed as units per 1 g protein (IU/g protein).

##### Glutathione Peroxidase (GPx) Activity (EC 1.11.1.9)

The GPx activity was measured using the kinetic method [36] with t-butyl peroxide as a substrate and expressed as μmoles of oxidized NADPH in 1 min per 1 g of protein (IU/g protein).

##### Glutathione Reductase (GR) Activity (EC 1.8.1.7)

The GR activity was determined using the kinetic method [37] and expressed in μmoles of NADPH utilized in 1 min by 1 g of protein (IU/g protein).

##### Glutathione-S Transferase (GST) Activity (EC 2.5.1.18)

The GST activity was estimated using the Habig and Jakoby kinetic method [38] and expressed as μmoles of thioether formed within 1 min per 1 g of protein (IU/g protein).

##### Sulfhydryl Groups (SH) Concentration

The concentration of the sulfhydryl groups (SH) (μmol/g protein) was determined with a semi-automatic method according to Koster et al. [39] and calculated from a calibration curve prepared with reduced glutathione as a standard.

##### Total Oxidant Status (TOS) and Total Antioxidant Capacity (TAC)

The total oxidant status (TOS) and total antioxidant capacity (TAC) levels were determined using the Erel methods [40,41].

##### Malondialdehyde (MDA) Concentration

The malondialdehyde (MDA) concentration was measured using a reaction with thiobarbituric acid [42], calculated from the standard curve prepared with 1,1,3,3-tetraethoxypropane, and expressed in μmol/g protein.

#### 4.4.4. Cholesterol, Triglycerides (TG), and HDL Concentrations

The cholesterol, TG, and HDL concentrations were measured using a BS-200E biochemical analyzer (Mindray, Shenzhen, China) and Alpha Diagnostics reagents (San Antonio, TX, USA).

### 4.5. Statistical Analysis

Statistical analyses were performed using Statistica v. 13.3.0 (TIBCO Software Inc., Palo Alto, CA, USA). All the tests were two-tailed, and the significance level was <0.05. The data distribution was assessed using the Shapiro–Wilk test and quantile–quantile plots. The non-normal data were presented as the median with lower and upper quartile (Me(Q_1_ − Q_3_). The intra-operative comparisons were performed using ANOVA and a non-parametric Kruskal–Wallis test with multiple comparisons. The data for the SHAM- and DJOS-operated animals were presented in one graph. The results for the different dietary pattern groups were connected with a dashed line to increase graph readability.

## 5. Conclusions

The results suggest that an HFS diet consumed before and after or only after DJOS surgery reduces the antioxidant potential of the liver. Nevertheless, DJOS surgery reduces serum lipids levels despite the type of diet used before and after the surgery. A lack of healthy habits after bariatric surgery in combination with an HFS diet may be responsible for DJOS treatment failure and lead to or establish an obesity condition including increased oxidative stress.

## Figures and Tables

**Table 1:** Cholesterol, triglyceride (TG), and HDL concentrations (mg/dL) in the serum of Sprague-Dawley rats (*n* = 56) after duodenojejunal omega switch (DJOS, *n* = 28,) or control (SHAM, *n* = 28) surgery; rats were fed according to different dietary patterns (CD/CD, *n* = 7; CD/HFS, *n* = 7; HFS/CD, *n* = 7; HFS/HFS, *n* = 7) for 8 weeks after the surgery.

Surgery Type	Parameter [mg/dL]	CD/CD	CD/HFS	HFS/CD	HFS/HFS	P_diet_
DJOS	Cholesterol	35 (33–37)	38 (37–39)	44 (44–45)	55 (54–56)	<0.001
	TG	49 (46–49)	57 (55–57)	54 (52–56)	59 (54–60)	<0.001
	HDL	16 (15–17)	20 (19–20)	23 (22–24)	20 (19–20)	<0.001
SHAM	Cholesterol	41 (40–41)	63 (63–65)	50 (50–50)	77 (74–79)	<0.001
	TG	61 (60–62)	135 (133–140)	75 (69–78)	149 (140–168)	<0.001
	HDL	11 (11–12)	13 (13–14)	36 (30–41)	12 (10–14)	<0.001

Legend: CD—control diet, DJOS—duodenojejunal omega switch surgery, HDL—high-density lipoprotein, HFS—high-fat, high-sugar diet, SHAM—control surgery.

**Table 2:** Comparison of cholesterol, triglyceride (TG), and HDL concentrations in the serum of Sprague-Dawley rats (*n* = 56) after duodenojejunal omega switch (DJOS, *n* = 28,) or control (SHAM, *n* = 28) surgery; rats were fed according to different dietary patterns (CD/CD, *n* = 7; CD/HFS, *n* = 7; HFS/CD, *n* = 7; HFS/HFS, *n* = 7) at 8 weeks after the surgery.

Surgery Type	Parameter	CD/CD vs. CD/HFS	CD/CD vs. HFS/CD	CD/CD vs. HFS/HFS	CD/HFS vs. HFS/CD	CD/HFS vs. HFS/HFS	HFS/CD vs. HFS/HFS
DJOS	Cholesterol	1	<0.05	<0.001	0.384	<0.01	0.668
	TG	<0.01	0.156	<0.001	1	1	0.690
	HDL	0.081	<0.001	0.106	0.149	1	0.115
SHAM	Cholesterol	<0.01	0.668	<0.001	0.688	0.688	<0.01
	TG	<0.01	0.668	<0.001	0.476	1	<0.05
	HDL	0.176	<0.001	1	0.284	0.917	<0.01

Legend: CD—control diet, DJOS—duodenojejunal omega switch surgery, HDL—high-density lipoprotein, HFS—high-fat, high-sugar diet, SHAM—control surgery.

## Data Availability

The research data are available upon reasonable request to the corresponding author.

## References

[1] Rani V., Deep G., Singh R.K., Palle K., Yadav U.C.S. (2016). Oxidative stress and metabolic disorders: Pathogenesis and therapeutic strategies. Life Sci..

[2] Świderska M., Maciejczyk M., Zalewska A., Pogorzelska J., Flisiak R., Chabowski A. (2019). Oxidative stress biomarkers in the serum and plasma of patients with non-alcoholic fatty liver disease (NAFLD). Can plasma AGE be a marker of NAFLD? Oxidative stress biomarkers in NAFLD patients. Free Radic. Res..

[3] Stefater M.A., Wilson-Perez H.E., Chambers A.P., Sandoval D.A., Seeley R.J. (2012). All bariatric surgeries are not created equal: Insights from mechanistic comparisons. Endocr. Rev..

[4] Stygar D., Sawczyn T., Skrzep-Poloczek B., Owczarek A.J., Matysiak N., Michalski M., Mielańczyk Ł., Bażanów B., Ziora P., Choręza P. (2018). The effects of duodenojejunal omega switch in combination with high-fat diet and control diet on incretins, body weight, and glucose tolerance in Sprague-Dawley rats. Obes Surg..

[5] Grueneberger J.M., Fritz T., Zhou C., Meyer S., Karcz-Socha I., Sawczyn T., Stygar D., Goos M., Hopt U.T., Küsters S. (2013). Long segment ileal transposition leads to early amelioration of glucose control in the diabetic obese Zucker rat. Videosurgery Other Miniinvasive Tech..

[6] Grueneberger J.M., Karcz-Socha I., Sawczyn T., Kosmowski J., Stygar D., Goos M., Küsters S., Zwirska-Korczala K., Marjanovic G., Keck T. (2014). Systematic ileal transposition in Zucker rats shows advantage for long segment distal transposition. Surgery.

[7] Stygar D., Skrzep-Poloczek B., Romuk E., Chełmecka E., Poloczek J., Sawczyn T., Maciarz J., Kukla M., Karcz K.W., Jochem J. (2019). The influence of high-fat, high-sugar diet and bariatric surgery on HSP70 and HSP90 plasma and liver concentrations in diet-induced obese rats. Cell Stress Chaperones.

[8] Rezzani R., Franco C. (2021). Liver, Oxidative stress and metabolic syndromes. Nutrients.

[9] Klisic A., Kavaric N., Ninic A., Kotur-Stevuljevic J. (2021). Oxidative stress and cardiometabolic biomarkers in patients with non-alcoholic fatty liver disease. Sci. Rep..

[10] Friedman S.L., Neuschwander-Tetri B.A., Rinella M., Sanyal A.J. (2018). Mechanisms of NAFLD development and therapeutic strategies. Nat. Med..

[11] Takaki A., Kawai D., Yamamoto K. (2013). Multiple hits, including oxidative stress, as pathogenesis and treatment target in non-alcoholic steatohepatitis (NASH). Int. J. Mol. Sci..

[12] Cichoż-Lach H., Michalak A. (2014). Oxidative stress as a crucial factor in liver diseases. World J. Gastroenterol..

[13] Morris E.M., Fletcher J.A., Thyfault J.P., Rector R.S. (2013). The role of angiotensin II in nonalcoholic steatohepatitis. Mol. Cell. Endocrinol..

[14] Wei Y., Clark S.E., Morris E.M., Thyfault J.P., Uptergrove G.M., Whaley-Connell A.T., Ferrario C.M., Sowers J.R., Ibdah J.A. (2008). Angiotensin II-induced non-alcoholic fatty liver disease is mediated by oxidative stress in transgenic TG(mRen2)27(Ren2) rats. J. Hepatol..

[15] Patrick L. (2002). Nonalcoholic fatty liver disease: Relationship to insulin sensitivity and oxidative stress. Treatment approaches using vitamin E, magnesium, and betaine. Altern. Med. Rev..

[16] Videla L.A. (2009). Oxidative stress signaling underlying liver disease and hepatoprotective mechanisms. World. J. Hepatol..

[17] Sumida Y., Niki E., Naito Y., Yoshikawa T. (2013). Involvement of free radicals and oxidative stress in NAFLD/NASH. Free Radic. Res..

[18] Ferro D., Baratta F., Pastori D., Cocomello N., Colantoni A., Angelico F., Del Ben M. (2020). New insights into the pathogenesis of non-alcoholic fatty liver disease: Gut-derived lipopolysaccharides and oxidative stress. Nutrients.

[19] Zhu R., Wang Y., Zhang L., Guo Q. (2012). Oxidative stress and liver disease. Hepatol. Res..

[20] Diesen D.L., Kuo P.C. (2010). Nitric oxide and redox regulation in the liver: Part I. General considerations and redox biology in hepatitis. J. Surg. Res..

[21] Muriel P. (2009). Role of free radicals in liver diseases. Hepatol. Int..

[22] Trefts E., Gannon M., Wasserman D.H. (2017). The Liver. Curr. Biol..

[23] Polimeni L., Del Ben M., Baratta F., Perri L., Albanese F., Pastori D., Violi F., Angelico F. (2015). Oxidative stress: New insights on the association of non-alcoholic fatty liver disease and atherosclerosis. World J. Hepatol..

[24] Brigelius-Flohé R., Maiorino M. (2013). Glutathione peroxidases. Biochim. Biophys. Acta.

[25] Gupta D.K., Palma J.M., Corpas F.J. (2016). Redox State as a Central Regulator of Plant-Cell Stress Responses.

[26] Geetha A., Lakshmi P., Jeyachristy S.A., Surendran R. (2007). Level of oxidative stress in the red blood cells of patients with liver cirrhosis. Indian J. Med. Res..

[27] Erel O. (2004). A novel automated direct measurement method for total antioxidant capacity using a new generation, more stable ABTS radical cation. Clin. Biochem..

[28] Marques S.S., Magalhães L.M., Tóth I.V., Segundo M.A. (2014). Insights on antioxidant assays for biological samples based on the reduction of copper complexes-the importance of analytical conditions. Int. J. Mol. Sci..

[29] Rubio C.P., Hernández-Ruiz J., Martinez-Subiela S., Tvarijonaviciute A., Ceron J.J. (2016). Spectrophotometric assays for total antioxidant capacity (TAC) in dog serum: An update. BMC Vet. Res..

[30] Furukawa S., Fujita T., Shimabukuro M., Iwaki M., Yamada Y., Nakajima Y., Nakayama O., Makishima M., Matsuda M., Shimomura I. (2004). Increased oxidative stress in obesity and its impact on metabolic syndrome. J. Clin. Investig..

[31] Masarone M., Rosato V., Dallio M., Gravina A.G., Aglitti A., Loguercio C., Federico A., Persico M. (2018). Role of oxidative stress in pathophysiology of nonalcoholic fatty liver disease. Oxid. Med. Cell. Longev..

[32] Russell W.M., Burch R.L. (1959). The Principles of Humane Experimental Technique.

[33] Lowry O.H., Rosebrough N.J., Farr A.L., Randall R.J. (1951). Protein measurement with the Folin phenol reagent. J. Biol. Chem..

[34] Oyanagui Y. (1984). Reevaluation of assay methods and establishment of kit for superoxide dismutase activity. Anal. Biochem..

[35] Aebi H. (1984). Catalase in vitro. Methods Enzymol..

[36] Mannervik B. (1985). Glutathione peroxidase. Methods Enzymol..

[37] Carlberg I., Mannervik B. (1985). Glutathione reductase. Methods Enzymol..

[38] Habig W.H., Jakoby W.B. (1981). Assays for differentiation of glutathione S-transferases. Methods Enzymol..

[39] Koster J.F., Biemond P., Swaak A.J. (1986). Intracellular and extracellular sulphydryl levels in rheumatoid arthritis. Ann. Rheum. Dis..

[40] Erel O. (2005). A new automated colorimetric method for measuring total oxidant status. Clin. Biochem..

[41] Erel O. (2004). A novel automated method to measure total antioxidant response against potent free radical reactions. Clin. Biochem..

[42] Ohkawa H., Ohishi N., Yagi K. (1979). Assay for lipid peroxides in animal tissues by thiobarbituric acid reaction. Anal. Biochem..

